# Analysis of key genes and pathways associated with the pathogenesis of intervertebral disc degeneration

**DOI:** 10.1186/s13018-020-01902-4

**Published:** 2020-09-01

**Authors:** Shiyu Hu, Yucheng Fu, Bin Yan, Zhe Shen, Tao Lan

**Affiliations:** 1 Department of Neurology, Shenzhen Second People’s Hospital, The First Affiliated Hospital of Shenzhen University, Shenzhen, China; 2 Department of Orthopedics, Ruijin Hospital Affiliated to Shanghai Jiaotong University School of Medicine, Shanghai, China; 3 Department of Spine Surgery, Shenzhen Second People’s Hospital, The First Affiliated Hospital of Shenzhen University, Shenzhen, China

**Keywords:** Intervertebral disc degeneration, Gene, Bioinformatics

## Abstract

**Background:**

Intervertebral disc degeneration (IDD) is widely known as the main contributor to low back pain which has a negative socioeconomic impact worldwide. However, the underlying mechanism remains unclear. This study aims to analyze the dataset GSE23130 using bioinformatics methods to identify the pivotal genes and pathways associated with IDD.

**Material/methods:**

The gene expression data of GSE23130 was downloaded, and differentially expressed genes (DEGs) were extracted from 8 samples and 15 controls. GO and KEGG pathway enrichment analyses were performed. Also, protein–protein interaction (PPI) network was constructed and visualized, followed by identification of hub genes and key module.

**Results:**

A total of 30 downregulated and 79 upregulated genes were identified. The DEGs were mainly enriched in the regulation of protein catabolic process, extracellular matrix organization, collagen fibril organization, and extracellular structure organization. Meanwhile, we found that most DEGs were primarily enriched in the PI3K-Akt signaling pathway. The top 10 hub genes were FN1, COL1A2, SPARC, COL3A1, CTGF, LUM, TIMP1, THBS2, COL5A2, and TGFB1.

**Conclusions:**

In summary, key candidate genes and pathways were identified by using integrated bioinformatics analysis, which may provide insights into the underlying mechanisms and offer potential target genes for the treatment of IDD.

## Introduction

Low back pain (LBP) is increasingly recognized as a global public health problem associated with decreased quality of life and increased healthcare expenditure [1–3]. It is estimated that 70–80% of the adult population suffer from at least one episode of LBP during their lifetime and 10% become chronically disabled [4]. With the changing of work and lifestyle, the incidence and prevalence of low back pain increase dramatically in the past few decades.

Intervertebral disc degeneration (IDD) is one of the most common sources of low back pain [5]. Being the largest avascular structure of the human body, the intervertebral disc is a complex structure consisting of the annulus fibrosus, nucleus pulposus, and cartilage endplate. The extracellular matrix (ECM) is the main component of the intervertebral disc which is responsible for maintaining both structure and function of the intervertebral disc. Intervertebral disc degeneration is characterized by the excessive degradation of ECM, leading to reduced hydration, loss of disc height, and decreased ability to absorb mechanical force [6].

Management of IDD includes both conservative treatment and surgery. Conservative management includes physiotherapy, medication, and epidural steroids injection. On the other hand, both fusion and non-fusion surgeries have demonstrated efficacy and safety in the management of IDD. The success rates of the Main and SPORT for surgically treated LDH patients and conservative-treated patients were 80% and 60%, respectively, at 1 year follow-up [7–9]. Although both methods have achieved satisfactory clinical outcomes, neither conservative nor surgical treatment can completely resolve lumbar disc degeneration. Hence, it is necessary to elucidate the underlying mechanisms of IDD to find out a curative method. Previous studies showed that IDD is most likely to be multifactorial, including apoptosis [10, 11], inflammation [12, 13], aging [14, 15], and biomechanical loading [16, 17]. However, the genetic factors are regarded as the most significant contributor [18].

Nowadays, the gene chip technology and bioinformatics methods have been widely used to obtain the gene expression profile of the disease. This study uses bioinformatics methods to analyze the microarray of the nucleus pulposus (GSE23130) with the aim to identify differentially expressed genes (DEGs) and pathways related to the progression of IDD. We also investigated some hub genes involved in the progression of IDD based on the protein–protein interaction (PPI) network. For example, MMP3 has been reported as a key gene in maintaining homeostasis of the extracellular matrix, and in vivo study showed that gene therapy targeting MMP3 was an efficient way to delay intervertebral disc degeneration [19]. Hence, to screen out DEGs as potential target candidates is of significance for the prevention and treatment of IDD. This study may provide new insights into the pathogenesis of IDD and potential target candidates for new therapy.

## Methods

### Microarray data collection

The gene expression data of GSE23130 was downloaded from the National Center of Biotechnology Information (NCBI) Gene Expression Omnibus (http://www.ncbi.nlm.nih.gov/geo/) database, which was based on the GPL1352 platform of Affymetrix Human X3P Array. Disc tissue samples were obtained either by the National Cancer Institute Cooperative Tissue Network (CHTN) or surgical disc procedures performed on patients with herniated discs and degenerative disc disease. Thompson grades IV and V are considered to be IDD samples while Thompson grades I, II, and III are control samples according to the Thompson grading criteria [20]. A total of 23 samples, including 15 control samples and 8 IDD samples, were contained in this dataset.

### Differential expression analysis

The Limma package version 3.28.21 (20) of Bioconductor 3.5 (http://www.bioconductor.org/packages/3.5/bioc/html/limma.html) was used to identify DEGs between the IDD samples and normal controls. Genes with fold change (logFC) > 1 (upregulated) or < − 1 (downregulated) and *P* value< 0.05 were considered differentially expressed. Both heatmap and volcano plots were constructed to present the expression profiles of differentially expressed genes using hierarchical clustering, which was performed using the R software.

### KEGG and GO enrichment analyses of DEGs

GO is a major bioinformatics tool to annotate genes and analyze gene products and sequences to underlying biological phenomena, including biological process (BP), molecular function (MF), and cellular component (CC) [21]. Kyoto Encyclopedia of Genes and Genomes (KEGG) is a knowledge base for systematic analysis, annotation, or visualization of gene functions and critical biological pathways closely related to intervertebral disc degeneration [22]. Both GO and KEGG enrichment analyses were conducted by Bioconductor. A *P* < 0.05 was considered to have statistical significance and to achieve significant enrichment.

### PPI network construction and analysis of modules

Protein–protein interaction (PPI) enrichment analysis is useful to analyze the functional interactions between proteins which may provide insights into the mechanisms of generation or development of diseases. Online String [23] and Cytoscape [24] software were used to build the PPI network, and a confidence score > 0.4 was set as the cutoff criterion. The most significant module was identified using Molecular Complex Detection (MCODE) using the following parameters: degree cutoff = 2, node score cutoff = 0.2, *k*-core = 2, and maximum depth = 100. GO and KEGG pathway enrichment analyses of genes in the most significant node were subsequently performed using String.

### Hub gene selection and analysis

CytoHubba was used to select hub genes of the PPI network with the Maximal Clique Centrality (MCC) method. The biological process of hub genes was then analyzed and visualized by the Biological Networks Gene Oncology tool (BiNGO; http://apps.cytoscape.org/apps/bingo) plugin.

## Results

### Differential expression analysis

**Fig. 1:**
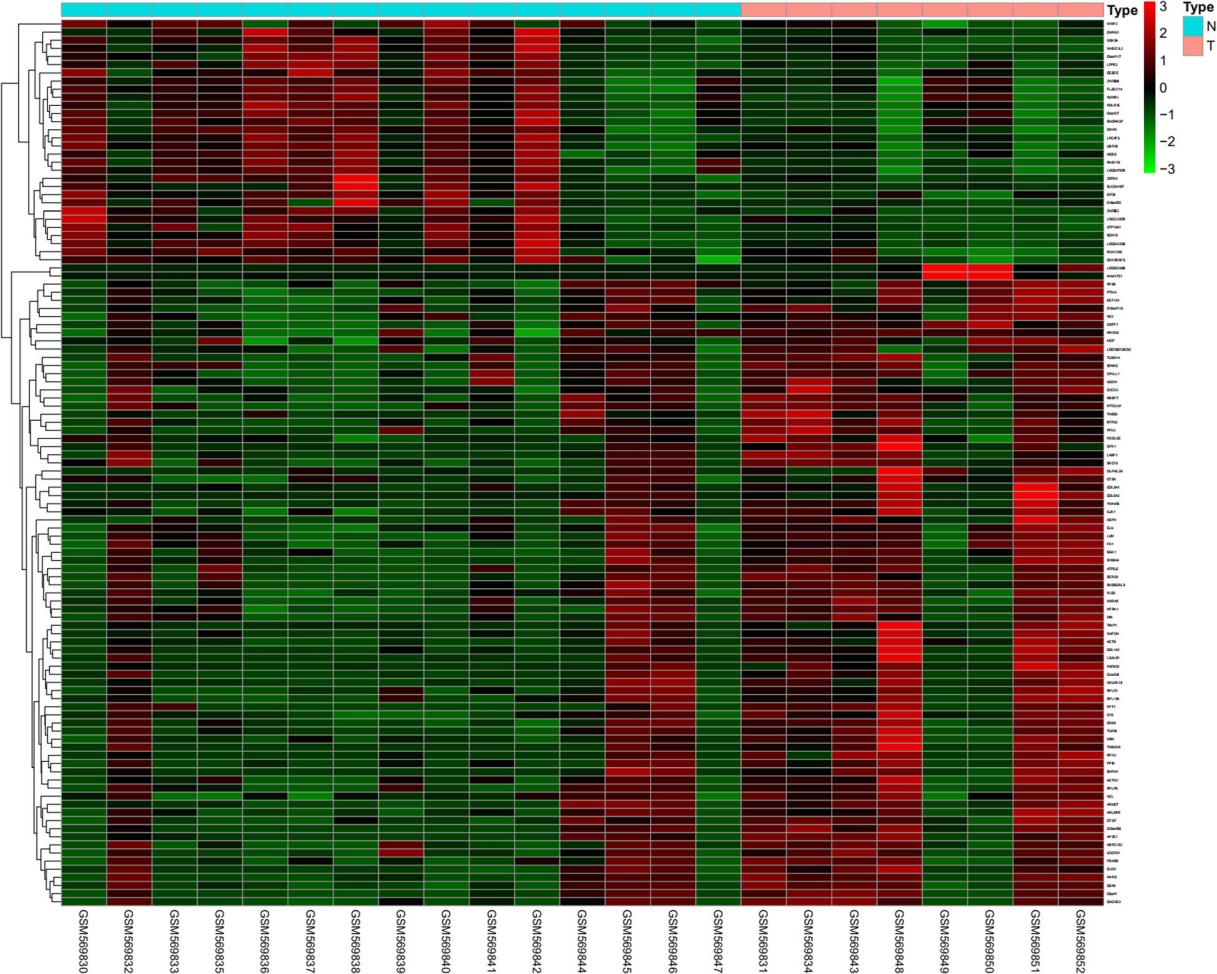
Heatmap plot of total DEGs

**Fig. 2:**
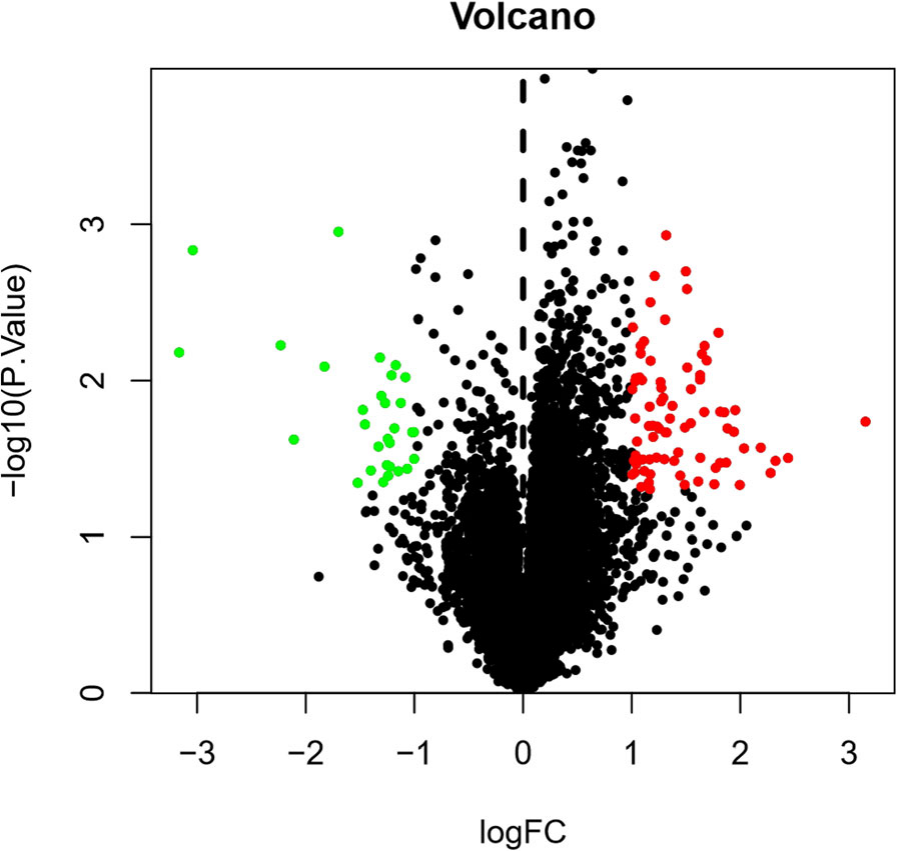
Volcano plot of total DEGs

A total of 19,703 DEGs were identified from intervertebral disc samples in GSE23130. Among them, 30 were downregulated and 79 were upregulated within the *P* value < 0.05 and |log2FC| > 1 criterion. A heatmap plot and a volcano plot are shown in Figs. 1 and 2, respectively, in which the red represents the upregulated genes and the green represents the downregulated genes. The top 10 upregulated and downregulated DEGs are shown in Table 1.

**Table 1:** The top 10 upregulated and downregulated DEGs

Group	Gene symbol	logFC	*P* value
Downregulated	DMKN	− 3.168	0.006602318
	LOC647070	− 3.042	0.001467741
	GSK3A	− 2.233	0.005937805
	FLJ32214	− 2.113	0.023887151
	ATP13A1	− 1.831	0.008133858
	RAB11B	− 1.702	0.001118076
	ZNF808	− 1.525	0.045232746
	USP28	− 1.478	0.015404176
	LOC642533	− 1.458	0.019098623
	LOC222070	− 1.403	0.03768916
Upregulated	LUM	3.147	0.018330652
	HTRA1	2.435	0.031358097
	SPARC	2.320	0.032710624
	RPLP0	2.275	0.03908414
	KIAA1751	2.184	0.026912864
	CLU	2.030	0.027190866
	ND2	1.991	0.046653239
	COL3A1	1.949	0.015460576
	S100A4	1.937	0.021301645
	MXRA5	1.877	0.020244629

### KEGG and GO enrichment analyses

**Fig. 3:**
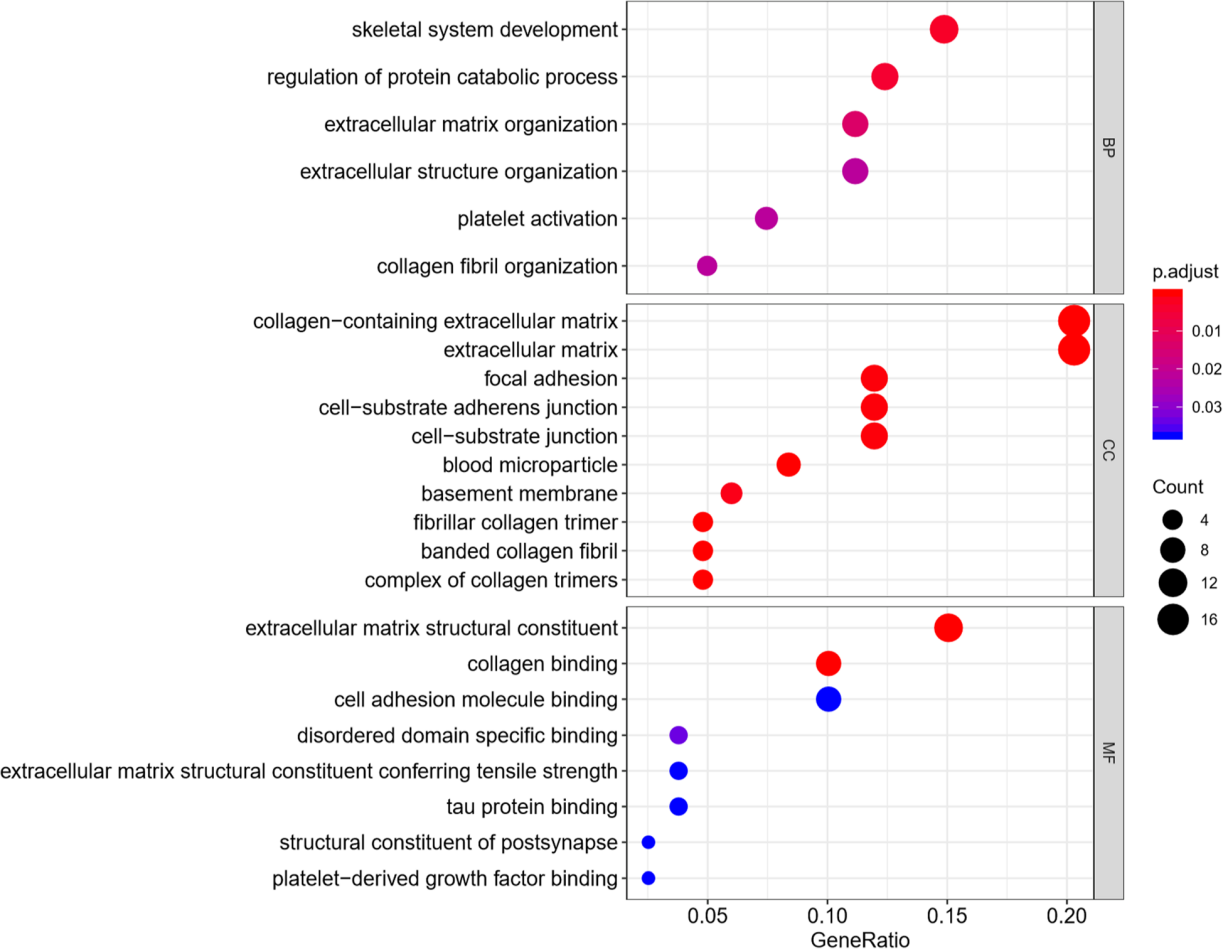
GO bubble chart of DEGs

**Fig. 4:**
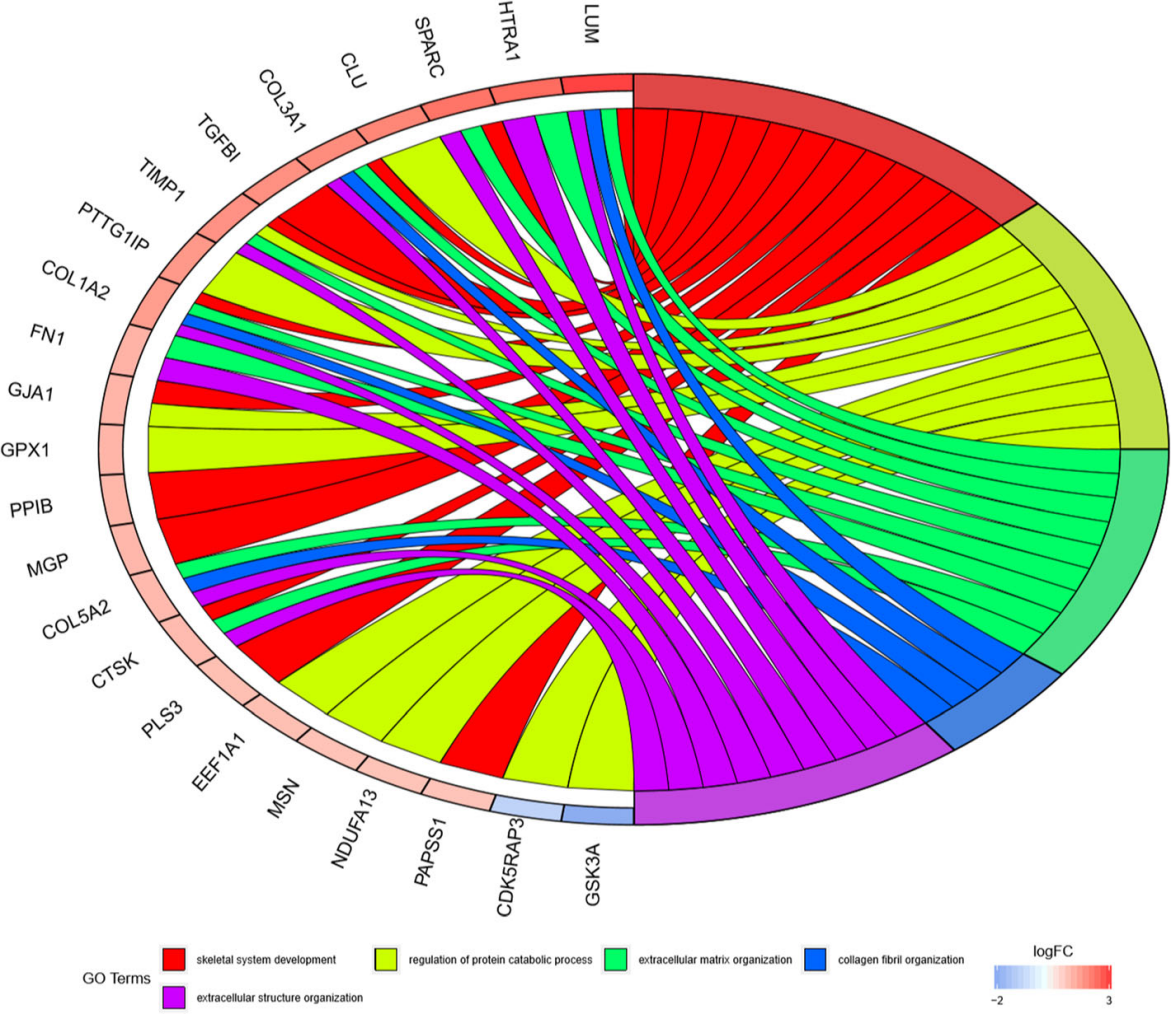
GO chord diagram of DEGs

**Fig. 5:**
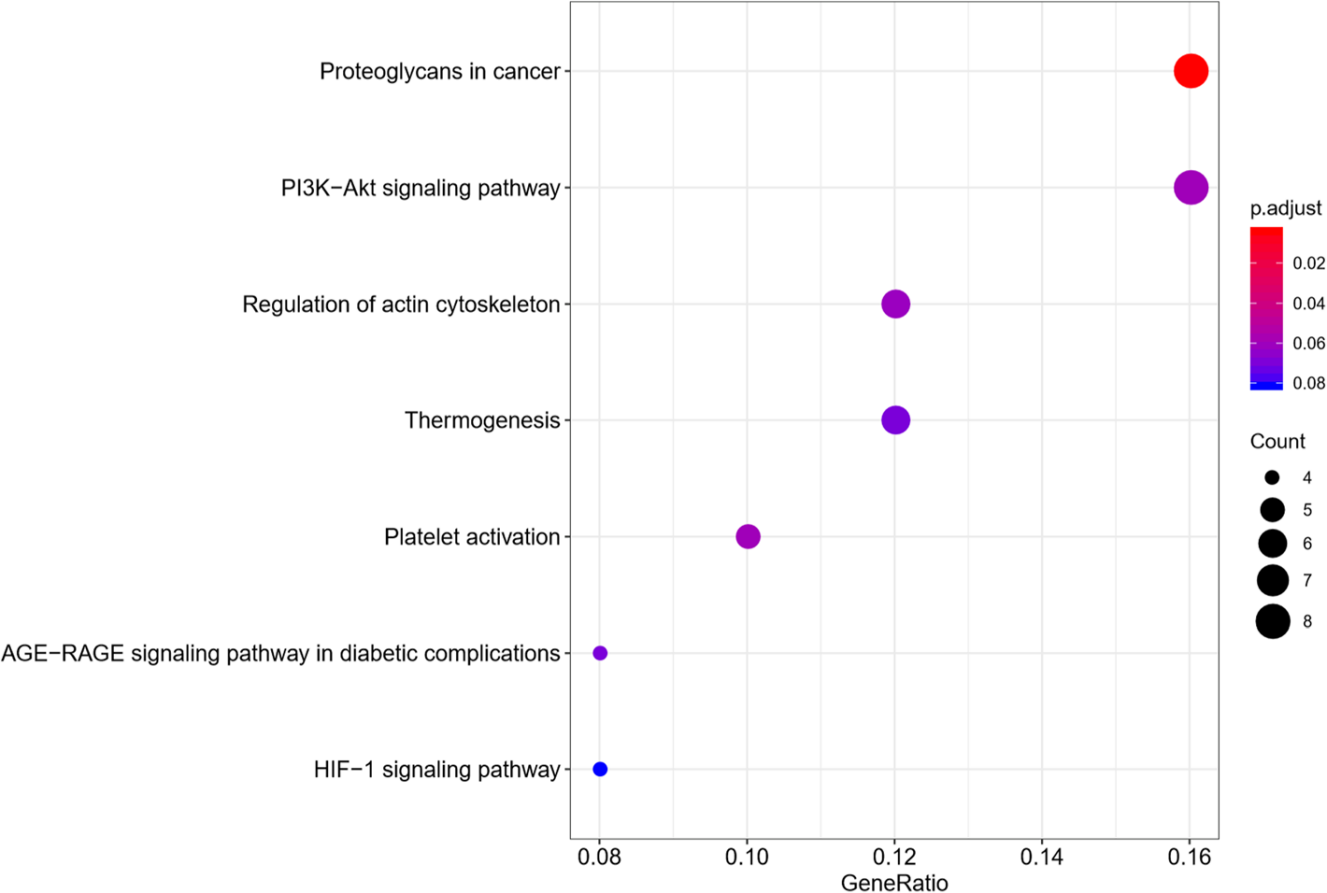
KEGG bubble chart of DEGs

**Fig. 6:**
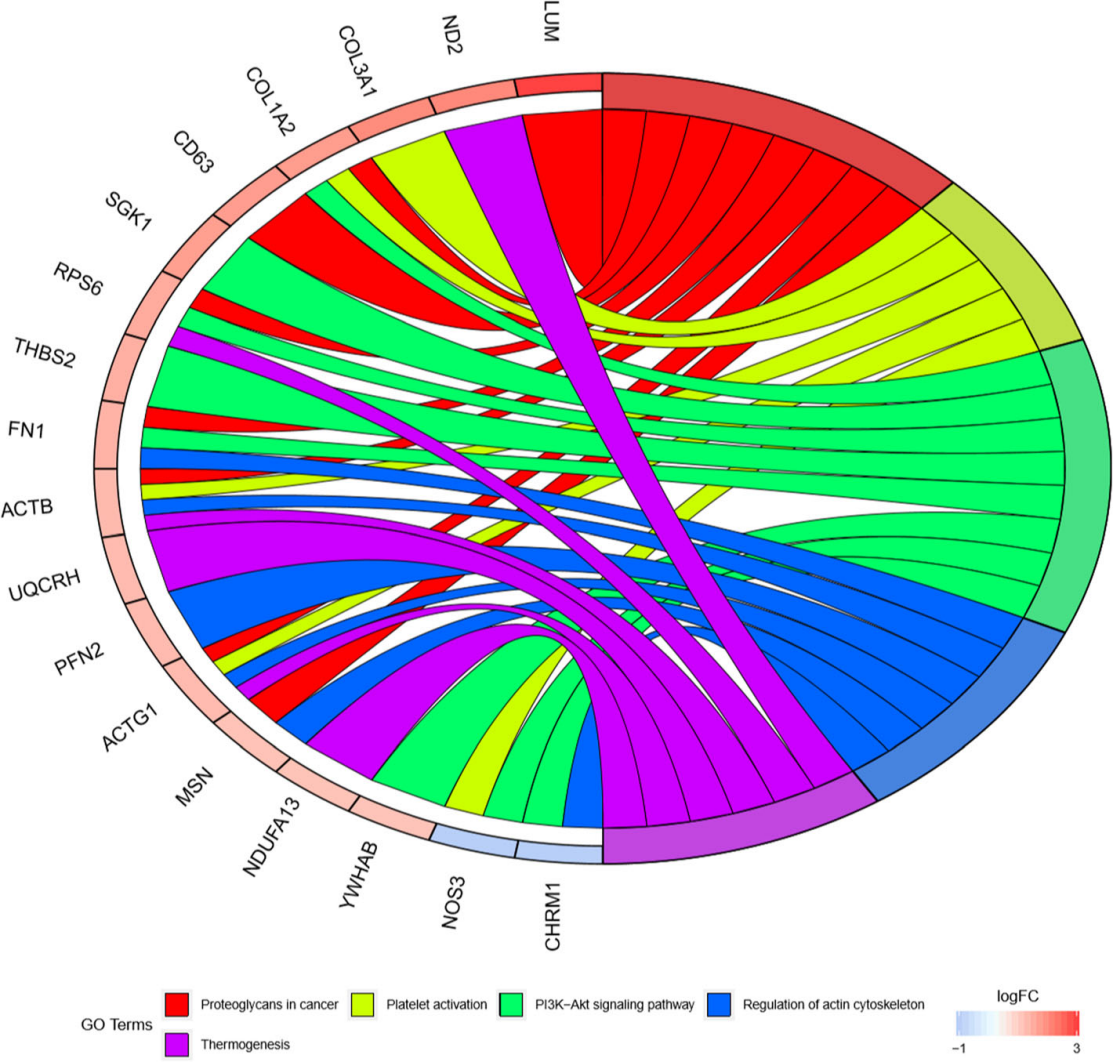
KEGG chord diagram of DEGs

We performed GO categories enrichment analysis to gain insights into the biological roles of the DEGs from degenerated versus non-degenerated disc samples. The DEGs were mainly enriched in skeletal system development, regulation of protein catabolic process, extracellular matrix organization, collagen fibril organization, and extracellular structure organization in terms of biological process (BP). The DEGs were mostly enriched in the collagen-containing extracellular matrix, extracellular matrix, fibrillar collagen trimer, banded collagen fibril, and complex of collagen trimers regarding cellular component (CC). The DEGs primarily participate in extracellular matrix structural constituent, collagen binding, disordered domain-specific binding, extracellular matrix structural constituent conferring tensile strength, and structural constituent of post-synapse concerning molecular function (MF). The top 5 BP, CC, and MF enrichment analyses of DEGs are summarized in Table 2. The GO enrichment-related bubble chart and circle plot are presented in Figs. 3 and 4. KEGG enrichment analysis showed that DEGs were enriched in “proteoglycans in cancer,” “platelet activation,” “PI3K-Akt signaling pathway,” “regulation of actin cytoskeleton,” and “thermogenesis.” The bubble chart and circle plot of KEGG enrichment were illustrated in Figs. 5 and 6.

**Table 2:** The top 5 BP, CC, and MF enrichment analyses of the upregulated and downregulated DEGs

Ontology	ID	Description	Gene ratio	*P* value	*P* adjust	Gene ID	Count
BP	GO:0001501	Skeletal system development	12/81	1.55E−06	0.003	COL1A2/PAPSS1/GJA1/COL3A1/TIMP1/LUM/CTSK/MGP/PPIB/PLS3/SPARC/TGFBI	12
BP	GO:0042176	Regulation of protein catabolic process	10/81	4.33E−06	0.004	GSK3A/MSN/EEF1A1/GJA1/GPX1/TIMP1/CLU/NDUFA13/CDK5RAP3/PTTG1IP	10
BP	GO:0030198	Extracellular matrix organization	9/81	1.96E−05	0.013	COL1A2/COL3A1/TIMP1/LUM/COL5A2/CTSK/FN1/HTRA1/SPARC	9
BP	GO:0030199	Collagen fibril organization	4/81	5.02E−05	0.021	COL1A2/COL3A1/LUM/COL5A2	4
BP	GO:0043062	Extracellular structure organization	9/81	6.18E−05	0.021	COL1A2/COL3A1/TIMP1/LUM/COL5A2/CTSK/FN1/HTRA1/SPARC	9
BP	GO:0030168	Platelet activation	6/81	6.50E−05	0.021	ACTB/COL1A2/NOS3/COL3A1/ACTG1/CLIC1	6
CC	GO:0062023	Collagen-containing extracellular matrix	17/84	1.98E−12	5.40E−10	COL1A2/PCOLCE/ASPN/LGALS1/COL3A1/TIMP1/THBS2/LUM/COL5A2/MGP/MXRA5/S100A4/FN1/CLU/HTRA1/SPARC/TGFBI	17
CC	GO:0031012	Extracellular matrix	17/84	2.47E−11	3.37E−09	COL1A2/PCOLCE/ASPN/LGALS1/COL3A1/TIMP1/THBS2/LUM/COL5A2/MGP/MXRA5/S100A4/FN1/CLU/HTRA1/SPARC/TGFBI	17
CC	GO:0005583	Fibrillar collagen trimer	4/84	1.23E−07	8.37E−06	COL1A2/COL3A1/LUM/COL5A2	4
CC	GO:0098643	Banded collagen fibril	4/84	1.23E−07	8.37E−06	COL1A2/COL3A1/LUM/COL5A2	4
CC	GO:0098644	Complex of collagen trimers	4/84	1.11E−06	6.06E−05	COL1A2/COL3A1/LUM/COL5A2	4
MF	GO:0005201	Extracellular matrix structural constituent	12/80	9.73E−12	2.35E−09	COL1A2/PCOLCE/ASPN/COL3A1/THBS2/LUM/COL5A2/MGP/MXRA5/FN1/SPARC/TGFBI	12
MF	GO:0005518	Collagen binding	8/80	4.10E−10	4.97E−08	PCOLCE/ASPN/LUM/CTSK/PPIB/FN1/SPARC/TGFBI	8
MF	GO:0097718	Disordered domain-specific binding	3/80	0.000412372	0.033	GJA1/FN1/GAPDH	3
MF	GO:0030020	Extracellular matrix structural constituent conferring tensile strength	3/80	0.000945361	0.037	COL1A2/COL3A1/COL5A2	3
MF	GO:0099186	Structural constituent of post-synapse	2/80	0.000963809	0.037	ACTB/ACTG1	2
KEGG	hsa05205	Proteoglycans in cancer	8/50	3.50E−05	0.004	ACTB/COL1A2/MSN/CD63/LUM/FN1/ACTG1/RPS6	8
KEGG	hsa04611	Platelet activation	5/50	0.001031369	0.059	ACTB/COL1A2/NOS3/COL3A1/ACTG1	5
KEGG	hsa04151	PI3K-Akt signaling pathway	8/50	0.001491165	0.059	YWHAB/COL1A2/SGK1/NOS3/THBS2/FN1/RPS6/CHRM1	8
KEGG	hsa04810	Regulation of actin cytoskeleton	6/50	0.00205303	0.061	ACTB/MSN/PFN2/FN1/ACTG1/CHRM1	6
KEGG	hsa04714	Thermogenesis	6/50	0.003083363	0.069	ACTB/ACTG1/NDUFA13/RPS6/ND2/UQCRH	6

### PPI network construction and analysis of modules

**Fig. 7:**
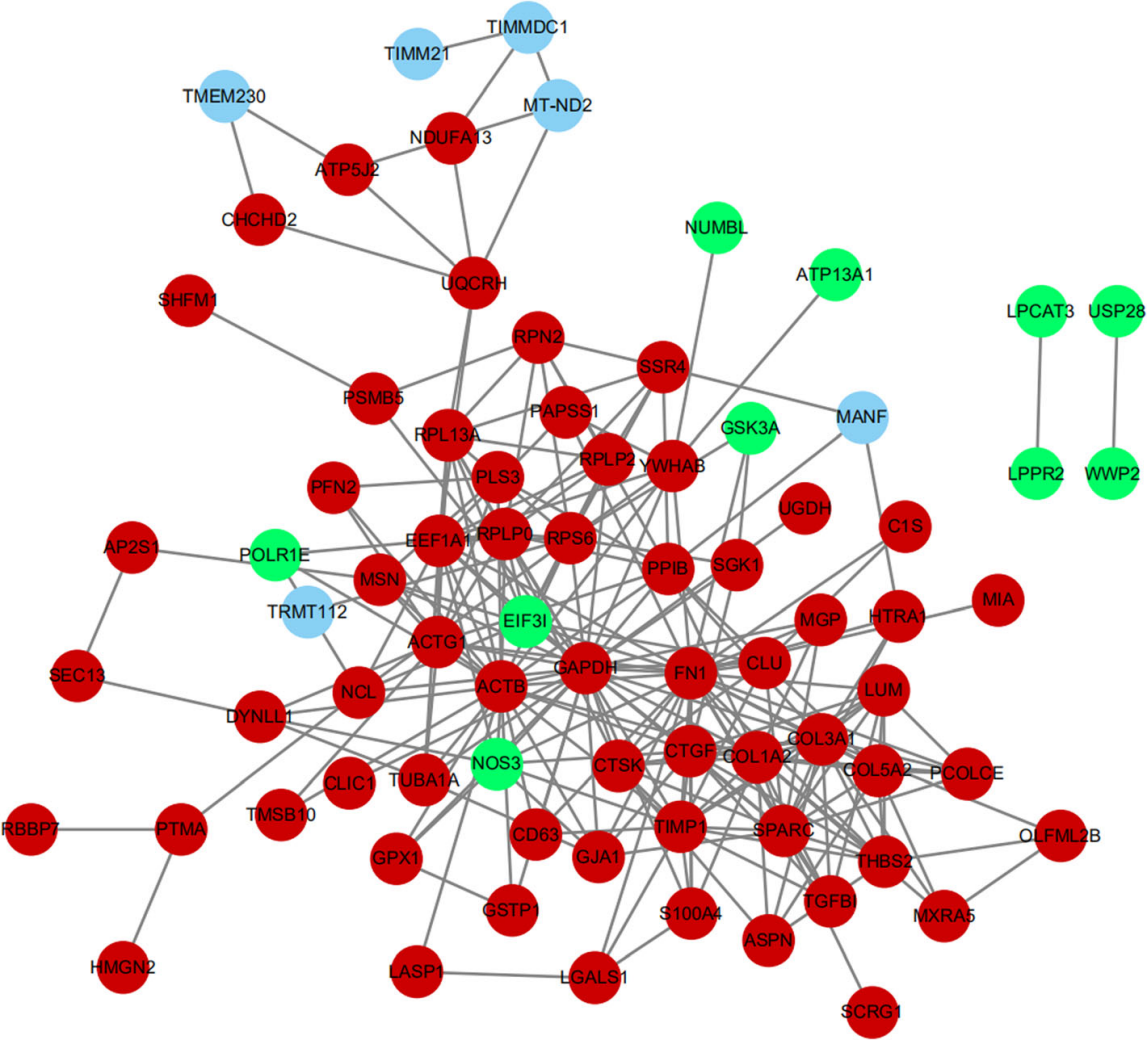
PPI network of DEGs

**Fig. 8:**
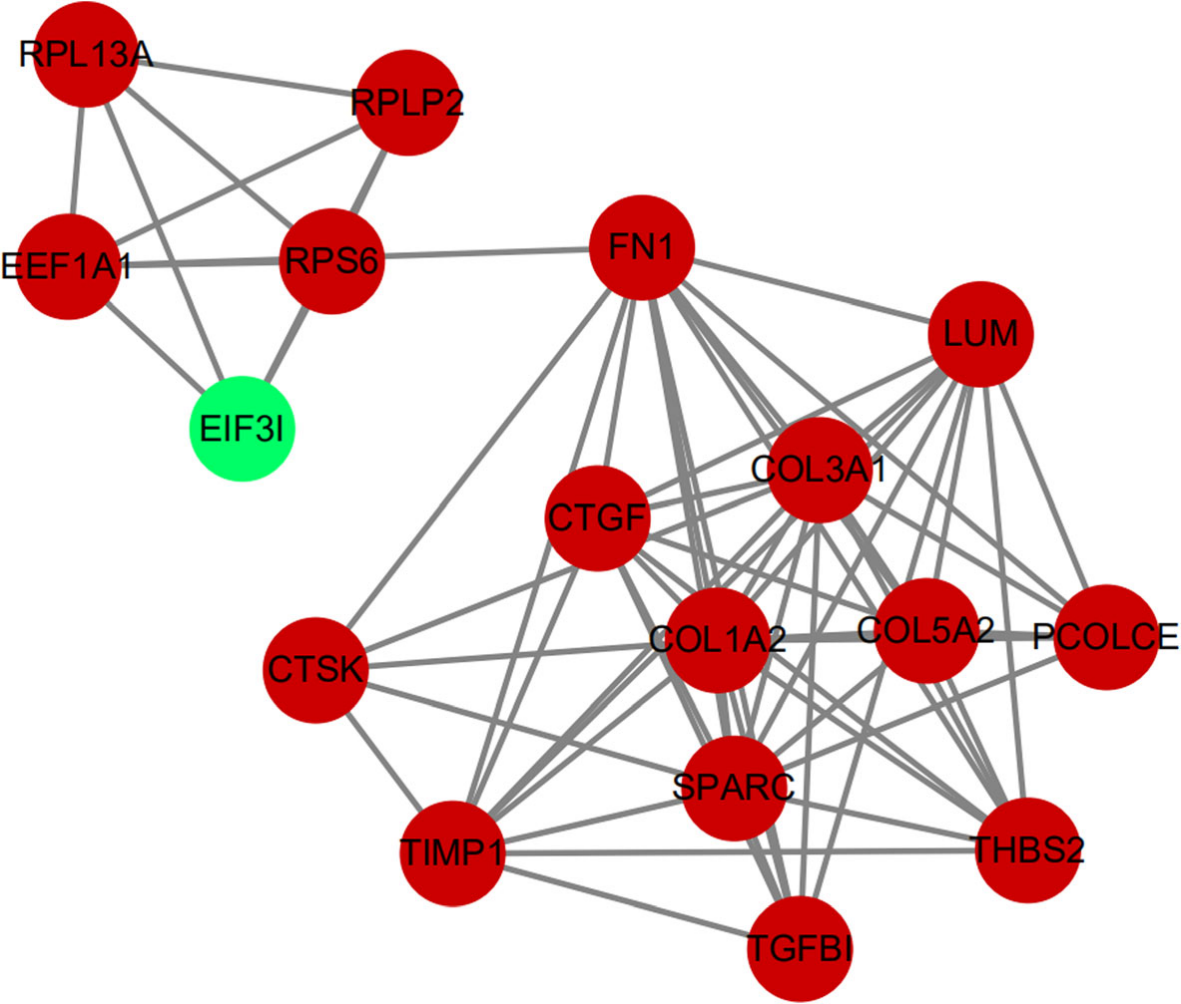
The most significant module

PPI network of DEGs was downloaded from String and further analyzed by Cytoscape. The PPI network included 110 nodes and 410 edges (Fig. 7). The most significant module was identified from the PPI network using MCODE and consisted of 17 nodes and 64 edges (Fig. 8). GO and KEGG enrichment analyses of this module using String showed that these genes were mainly involved in extracellular matrix organization, skeletal system development, protein digestion and absorption, and ribosome.

### Hub gene selection and analysis

**Fig. 9:**
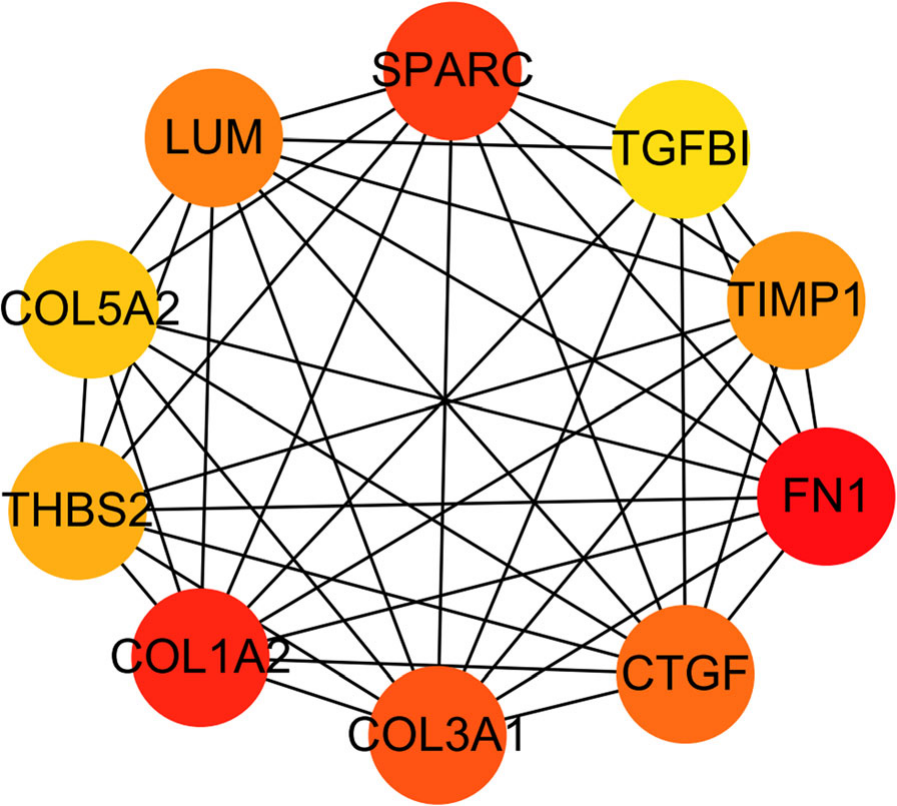
The top 10 hub genes

The top 10 hub genes were identified by CytoHubba plugin using the Maximal Clique Centrality (MCC) method, including FN1, COL1A2, SPARC, COL3A1, CTGF, LUM, TIMP1, THBS2, COL5A2, and TGFB1 (Fig. 9). All hub genes were upregulated in the degenerated disc compared with the control group. Analysis of hub genes was summarized in Table 3.

**Table 3:** The top 10 hub genes

Rank	Name	Score	Degree
1	FN1	18070	10
2	COL1A2	17598	18
3	SPARC	17455	17
4	COL3A1	17338	17
5	CTGF	16346	15
6	LUM	15840	10
7	TIMP1	12030	16
8	THBS2	10106	10
9	COL5A2	5796	11
10	TGFBI	5040	7

## Discussion

Despite years of numerous clinical and experimental investigations, the underlying mechanisms of intervertebral disc degeneration remain unclear, which hinders the development of curative therapy. Genetic factors, mechanical factors, aging, inflammation, and other potential factors may cause IDD whereas genetic factors play a critical role based on published literatures. Several studies indicate that genetic factors are critical contributors to the onset and progression of IDD [25, 26]. For example, COL1A1 is a key gene encoding collagen I, and polymorphisms of the COL1A1 gene have been reported to increase the risk of IDD in different population studies [27, 28]. In the present study, we identify a total of 109 DEGs between degenerative samples and controls, including 79 upregulated and 30 downregulated DEGs.

In terms of GO enrichment analysis, we found that most of DEGs were mainly involved in skeletal system development, regulation of protein catabolic process, extracellular matrix (ECM) organization, collagen fibril organization, and extracellular structure organization. The extracellular matrix (ECM) is a non-cellular three-dimensional macromolecular network predominantly composed of collagens, proteoglycans, and many other glycoproteins. ECM is crucial for maintaining the structural and functional integrity of the intervertebral disc. Previous studies showed that even though many potential mechanisms induced intervertebral disc (IVD), they led to a final common result of excessive degradation of the extracellular matrix [29]. The imbalance between anabolism and catabolism of ECM is regulated by ECM-modifying enzymes such as matrix metalloproteinases (MMPs) and their endogenous tissue inhibitors of metalloproteinases (TIMPs) [30–32]. Lumican (LUM) is the most significantly upregulated gene in our analysis, which is one kind of keratan sulfate proteoglycan constituents of the ECM. Several studies showed that the abundance of lumican changed with the degeneration of the intervertebral disc [33, 34]. The study conducted by Vo et al. showed that ECM degradation increased by regulation of matrix metalloproteinases (MMPs) and ADAMTSs, leading to the development of IDD [35]. On the contrary, TIMP-1 and TIMP-2 mRNA and protein expression increase in degenerated IVD tissue, antagonizing the effect of MMPs [36]. Our bioinformatics analysis also showed that TIMP-1 increased in the IVD samples than controls.

Regarding the KEGG pathway of our analysis, we found that most DEGs were primarily enriched in the PI3K-Akt signaling pathway. The PI3K-Akt signal pathway showed protective effects on the human nucleus pulposus under different pathological conditions. Activation of the PI3K-Akt pathway protects against IDD by the increase of ECM content, prevention of cell apoptosis, and induction or prevention of cell autophagy. Studies conformed that the activation of the PI3K-Akt pathway increased the SOX9 expression and activity and consequently led to the increase of the aggrecan expression in NP cells [37]. A study revealed that 17β-estradiol (E2) prevented the degradation of ECM by the activation of PI3K-Akt-FOXO3, which reduced the expression of MMP-3 and increased the expression of collagen II and aggrecan expression [38]. Many recent studies also showed that resveratrol suppressed IL-1β-mediated NP cell apoptosis through activating the PI3K-Akt pathway [39–41]. On the contrary, as the only known lipid phosphatase, tumor suppressor phosphatase and tensin homolog deleted from chromosome 10 (PTEN) can counteract the protective effect of the PI3K-Akt pathway. Xi et al. showed that PTEN promoted intervertebral disc degeneration by negatively influence PI3K-Akt [42]. Hence, gene therapy targeting PTEN may play an important role in treating IDD.

We further constructed a PPI network for better understanding of the interaction between DEGs. The most significant module was extracted from the PPI network using the MCODE plugin. Furthermore, the top 10 hub genes—FN1, COL1A2, SPARC, COL3A1, CTGF, LUM, TIMP1, THBS2, COL5A2, and TGFBI—were identified from this network. To be mentioned, all hub genes were also enriched in the most significant module. TGFBI is the seed DEG of the module. TGFBI is a protein secreted by many types of cells. It binds to collagen, forms part of the extracellular matrix (ECM), and interacts with integrins on cell surfaces. The study showed that TGF-β increased the expression of COL1A1, ACAN, and SOX9 genes by mediating communication between nucleus pulposus cells and mesenchymal stem cells [43]. Activation of TGF-β signaling has a protective effect on the intervertebral disc via inhibition of ECM degradation and increase of ECM synthesis, promotion of cell proliferation and inhibition of cell death, and alleviation of inflammatory response. However, excessive activation of TGF-β signaling may contribute to IVD degeneration [44]. SPARC is a matricellular glycoprotein involved in interactions between cells and matrices. Gruber showed that the deletion of the SPARC gene accelerated disc degeneration in the aging mouse [45]. Millecamps et al. demonstrated that inactivation of the SPARC gene led to the early onset of both disc degeneration and behavioral indices of LBP in mice [46]. Tajerian et al. showed that the underlying mechanism of the silence of the SPARC gene during aging may be attributed to DNA methylation [47]. A recent in vivo experimental study showed that stable expression of CTGF and TIMP1 genes by co-transfection adeno-associated virus 2 increased synthesis of aggrecan and type II collagen in the degenerated intervertebral disc, which served as a potential target gene for disc regeneration [48]. Thrombospondin proteins (THBSs) are a class of glycoproteins that function in maintaining homeostasis of ECM by regulating the level of matrix metalloproteinase-2 (MMP-2) and MMP-9 [49, 50]. A Japanese population-based genetic and functional data indicated that THBS2 played an important role in the pathogenesis of LDH by acting as a modulator of MMP-2 and MMP-9 endocytosis [51].

## Conclusion

In summary, the present study provides a comprehensive analysis about the pathogenesis of IDD and offers potential target genes for the early diagnosis and treatment of intervertebral disc disease. The PI3K-Akt signal pathway and related hub genes may play important roles in the progression of IDD that needs deeper investigation. Nevertheless, further experiments are required to validate their effects and mechanisms in IDD.

## Data Availability

The following information was supplied regarding data availability. The raw data can be found at https://www.ncbi.nlm.nih.gov/geo/query/acc.cgi?acc=GSE23130.

## Abbreviations

LBP: Low back pain
IVD: Intervertebral disc
IDD: Intervertebral disc degeneration
AF: Annulus fibrosus
NP: Nucleus pulposus
CEP: Cartilage endplate
miRNA: MicroRNA
ECM: Extracellular matrix
MMP: Matrix metalloproteinase
DEGs: Differentially expressed genes
BP: Biological process
CC: Cellular component
MF: Molecular function
KEGG: Kyoto Encyclopedia of Genes and Genomes
